# The risk of pancreatic adenocarcinoma following SARS-CoV family infection

**DOI:** 10.1038/s41598-021-92068-4

**Published:** 2021-06-21

**Authors:** Amin Ebrahimi Sadrabadi, Ahmad Bereimipour, Arsalan Jalili, Mazaher Gholipurmalekabadi, Behrouz Farhadihosseinabadi, Alexander M. Seifalian

**Affiliations:** 1 Department of Stem Cells and Developmental Biology at Cell Science Research Centre, Royan Institute, Tehran, Iran; 2 Faculty of Sciences and Advanced Technologies in Biology, University of Science and Culture, Tehran, Iran; 3 Parvaz Research Ideas Supporter Institute, Tehran, Iran; 4 Cellular and Molecular Research Centre, Department of Tissue Engineering and Regenerative Medicine, Iran University of Medical Sciences, Tehran, Iran; 5 Department of Medical Biotechnology, Iran University of Medical Sciences, Tehran, Iran; 6 Hematopoetic Stem Cell Research Centre, Shahid Beheshti University of Medical Sciences, Tehran, Iran; 7 Nanotechnology and Regenerative Medicine Commercialization Centre (Ltd), London BioScience Innovation Centre, London, UK

**Keywords:** Viral infection, Cancer

## Abstract

COVID 19 disease has become a global catastrophe over the past year that has claimed the lives of over two million people around the world. Despite the introduction of vaccines against the disease, there is still a long way to completely eradicate it. There are concerns about the complications following infection with SARS-CoV-2. This research aimed to evaluate the possible correlation between infection with SARS-CoV viruses and cancer in an in-silico study model. To do this, the relevent dataset was selected from GEO database. Identification of differentially expressed genes among defined groups including SARS-CoV, SARS-dORF6, SARS-BatSRBD, and H1N1 were screened where the |Log FC| ≥ 1and *p* < 0.05 were considered statistically significant. Later, the pathway enrichment analysis and gene ontology (GO) were used by Enrichr and Shiny GO databases. Evaluation with STRING online was applied to predict the functional interactions of proteins, followed by Cytoscape analysis to identify the master genes. Finally, analysis with GEPIA2 server was carried out to reveal the possible correlation between candidate genes and cancer development. The results showed that the main molecular function of up- and down-regulated genes was “double-stranded RNA binding” and actin-binding, respectively. STRING and Cytoscape analysis presented four genes, *PTEN, CREB1, CASP3*, and *SMAD3* as the key genes involved in cancer development. According to TCGA database results, these four genes were up-regulated notably in pancreatic adenocarcinoma. Our findings suggest that pancreatic adenocarcinoma is the most probably malignancy happening after infection with SARS-CoV family.

## Introduction

As of the late December 2020 over 200 million new Covid-19 cases have been reported, with more than 1,750,000 deaths worldwide^1^. The full clinical manifestations are not yet known, as the reported symptoms vary from mild to severe and may lead to death. Fever, cough, fatigue, pneumonia, headache, and severe shortness of breath are the most commonly reported symptoms. Nausea, diarrhea, hemoptysis, runny nose, and phlegm cough are less common. Patients with mild symptoms are reported to recover after one week, while in severe cases, due to virus-induced alveolar damage, they experience progressive respiratory failure which may lead to death^2^. In March 2020, the World Health Organization (WHO) identified Covid-19 as a pandemic and called on governments around the world to manage the protection of the population against COVID-19^3,4^. Considering the wide range of infections with this virus and given that lack of information regarding the pathogenesis and even transmission, there have been concerns about the consequences following infection with SARS-CoV-2 virus. Therefore, further researches in this field can be very valuable to predict diseases such as malignancies that may occur after infection with the virus in the long term.

Until now, the role of viruses in many types of cancer has been proven. For example, the association between human papillomavirus infection and cervical cancer has been extensively studied^5^. Different mechanisms have been proposed to justify the tumorigenesis of viruses. For example, degrading vital cell oncogenes through binding to virus proteins is one of the pathways involved in the tumorigenesis of viruses^6^. In addition, viruses may induce genomic instability and alter the expression levels of vital cell-regulating molecules such as miRNAs. In SARS-COV-1 infection, changes in the quantity and quality of tumour suppressor proteins such as pRb have been reported. It is found that SARS-COV-1 Nsp 15 down-regulates the expression of pRb and promotes its degradation via the proteosome-ubiquitin pathway^7^. Moreover, loss of cell–cell contact inhibition occurs following SARS-CoV virus infection^8^. Oxidative stress is another mechanism that may lead to carcinogenesis after viral infections. In fact, inflammatory response, cytokine storm, and oxidative stress are considered as the main cause of acute respiratory distress syndrome in patient with SARS-CoV virus infection. ROS production following oxidative stress has been identified as a trigger of carcinogenesis through single-stranded and double-stranded DNA breakage, DNA cross-linking, and inhibition of mismatch repair^9^. In addition, ROS can enhance cell invasion, proliferation, angiogenesis, cell survival, and even drug resistance by interacting with intracellular signalling pathways, indicating the possible role of these molecules in cancer development following viral infections^10^.

The emerging of high-throughput technologies and computational frameworks lead to develop a new field of medicine these years that allows researchers to freely study different biological systems^11–13^. Network-based approaches cover a wide range of medicine branches from personal medicine to cancer diagnosis^14,15^. With the help of graphical networks of complex biological systems, researchers are able to understand how a cluster of genes in a group of signalling pathways are involved in response to a special drug, infection, disease, and etc^16,17^. This new discipline helps to raise new diagnostic or therapeutic possibilities in the event that a new disease has emerged^18–20^. Beside this, it helps to identify the complications that may occur after exposure to an infectious disease.

As mentioned earlier, there is no reliable experimental data on the carcinogenicity of SARS-COV-2 virus. Most of studies have focused on the virus' ability to cause respiratory distress. However, this should not stop further research into the possibility that the virus may cause other types of disease. There is an increasing interest in employing enrichment dataset and in silico functional annotation analysis. This in silico-based analysis provides a well-defined hypothesis and rational concept, which shed light on experiment design. To date, no study has visualized the possible association between COVID-19 infection and any form of malignancy development. Moreover, there are no categorized genes and signalling pathways with a typical regulatory role in both COVID-19 and cancer development. Therefore, it would be interesting to investigate these signalling pathways and reveal its possible mechanistic relationship. Based on this fact, we raise the hypothesis that COVID-19 infection may induce cancer-related signalling pathways and regulate the downstream genes which can develop cancer in the long term.

## Results

### Identification and characteristics of up/down-regulated DEGs

**Figure 1:**
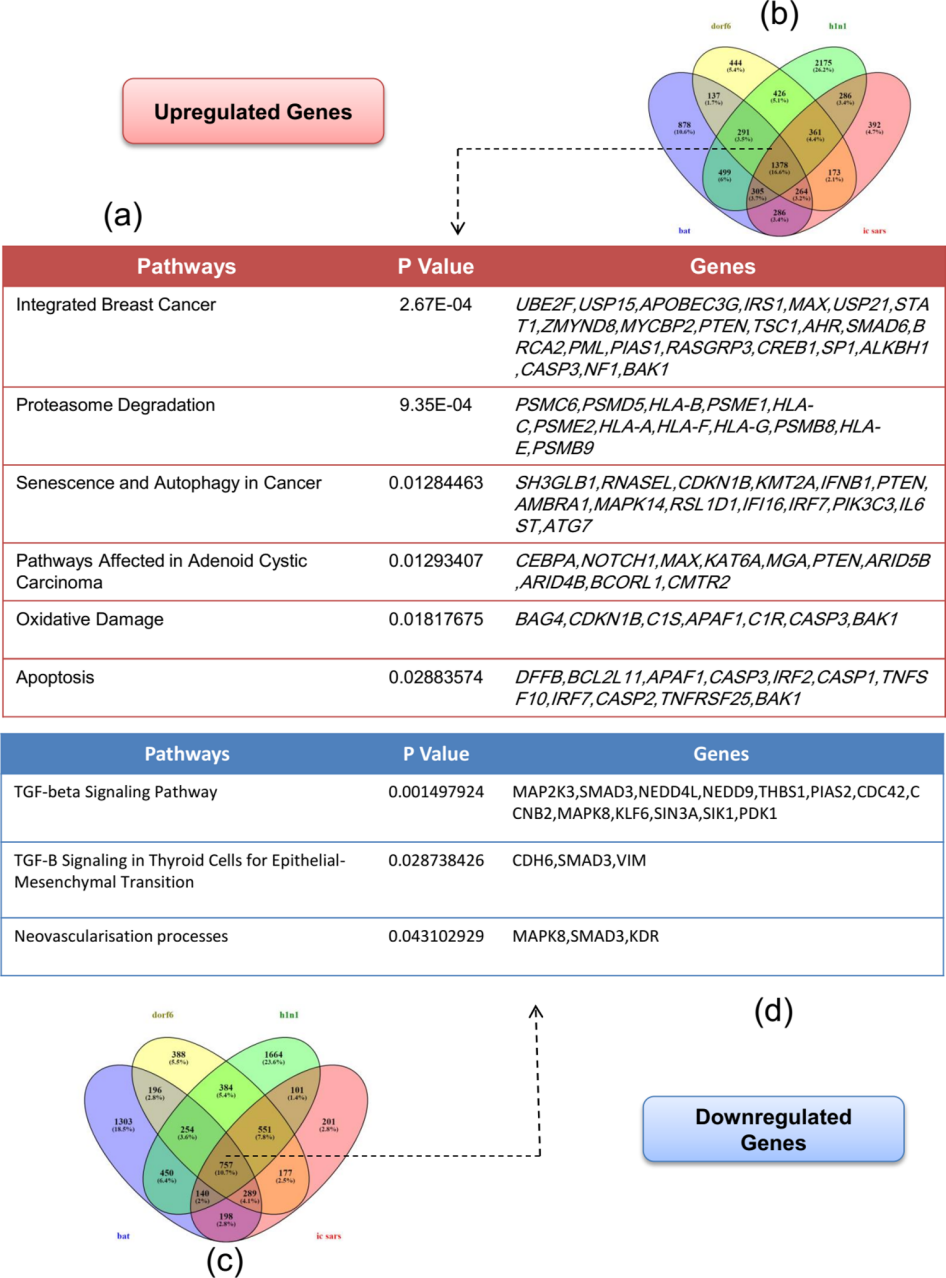
The cancer-related up/down-regulated common DEGs filtered based on *p* value < 0.05. (**a**) The upregulated DEGs. (**b**) The Venn diagram of the common upregulated DEGs between four groups. Both up-regulated and down-regulated DEGs has been filtered based on *p* value < 0.05 and involvement in cancer pathways. (**c**) The Venn diagram of the common down-regulated DEGs between four groups (**d**) The down regulated DEGs.

The Venn diagram of four defined groups (SARS-CoV, SARS-dORF6, SARS-BatSRBD, and H1N1) has been visualized. Between the total number of genes that were measured through the GPL6480 platform, 392 (SARS-CoV), 444 (SARS-dORF6), 875 (SARS-BatSRBD), and 2175 (H1N1) genes showed upregulation (Fig. 1a). Downregulation of 201 (SARS-CoV), 388 (SARS-dORF6), 1303 (SARS-BatSRBD), and 1664 (H1N1) genes have been categorized (Fig. 1b). The DEGs between all groups were 1378 (up-regulated) and 757 (downregulated). The up/downregulated DEGs have been filtered based on their role in cancer-related signalling pathways aim to highlight the most statistically significant cancer-related genes. Therefore, 5% (78) of the up-regulated DEGs (Fig. 1c) and 2% (19) of the down-regulated DEGs (Fig. 1d) were specified as the most significant cancer-related DEGs in all four groups.

### Biological process and molecular functional of enrichment analysis

**Figure 2:**
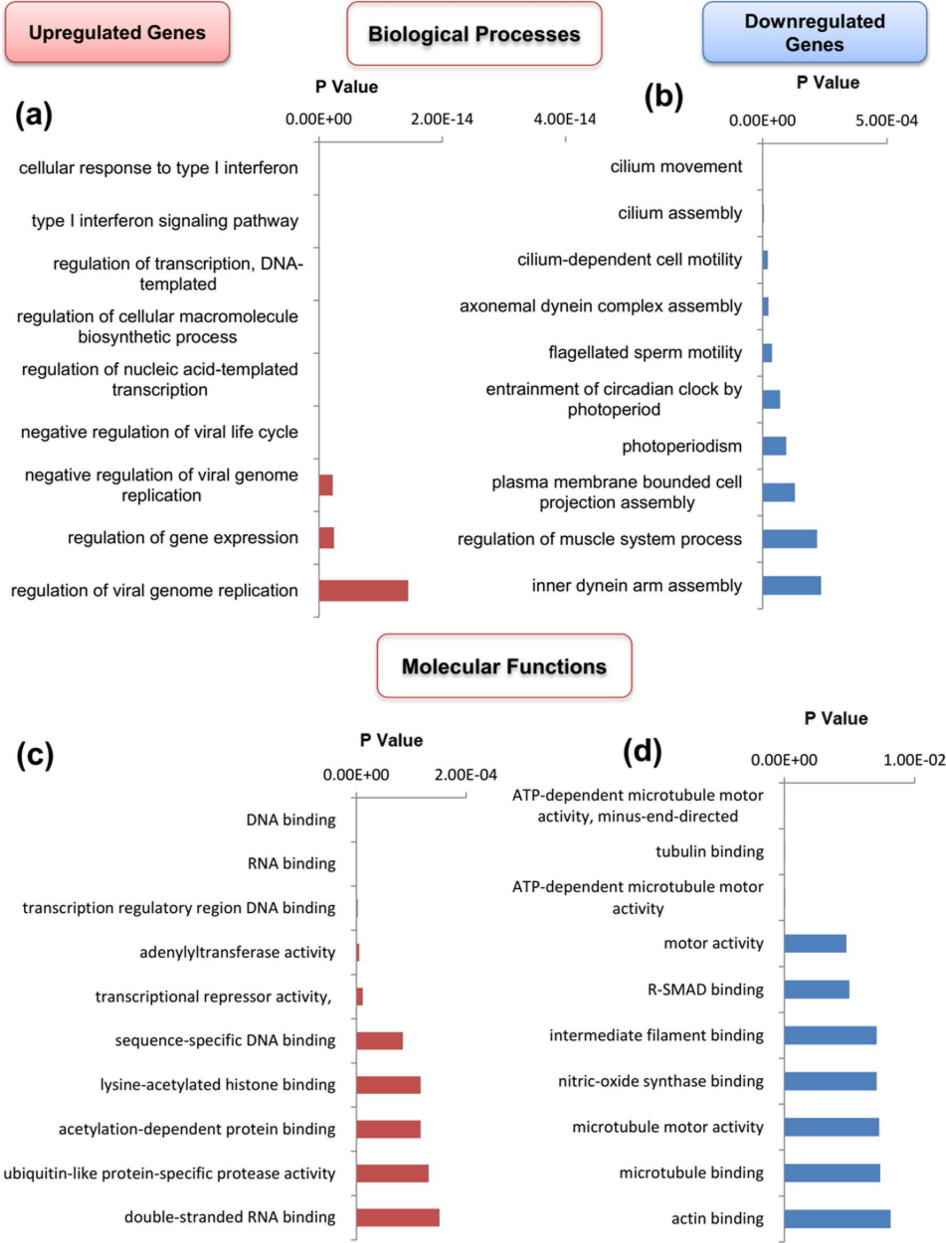
The biological process and molecular function of up/down-regulated DEGs. (**a**) The biological process of up-regulated genes. (**b**) The biological process of down-regulated genes. (**c**) The molecular function of up-regulated genes. (**d**) The molecular function of down-regulated genes.

Enrichr tool analysis showed that the up-regulated DEGs were enriched in 19 GO terms, while down-regulated DEGs were incorporated with 20 GO terms. Functional enrichment analysis showed that the biological process (BP) term “regulation of viral genome replication” (GO:0045069) (*p* < 0.0001) was significantly overexpressed in up-regulated DEGs (Fig. 2a). BP investigation of down-regulated DEGs highlighted “plasma membrane bounded cell projection assembly” (GO:0120031) (*p* < 0.0001), “regulation of muscle system process” (GO:0090257) (*p* < 0.001), and “inner dynein arm assembly” (GO:0036159) (*p* < 0.001) (Fig. 2b).

Overrepresented molecular function (MF) terms in up-regulated DEGs included “double-stranded RNA binding" (GO:0003725) (*p* < 0.001), "ubiquitin-like protein-specific protease activity" (GO:0019783) (*p* < 0.001), "acetylation-dependent protein binding" (GO:0140033) (*p* < 0.001), and "lysine-acetylated histone binding" (GO:0070577) (*p* < 0.001) (Fig. 2c). Interestingly, MF analysis of down-regulated DEGs showed an overexpression in "actin binding" (GO:0003779) (*p* < 0.01), "microtubule binding" (GO:0008017) (*p* < 0.01), "microtubule motor activity" (GO:0003777) (*p* < 0.01), "nitric-oxide synthase binding" (GO:0050998) (*p* < 0.01), and "intermediate filament binding" (GO:0019215) (*p* < 0.01) (Fig. 2d).

### PPI visualization of DEGs

**Figure 3:**
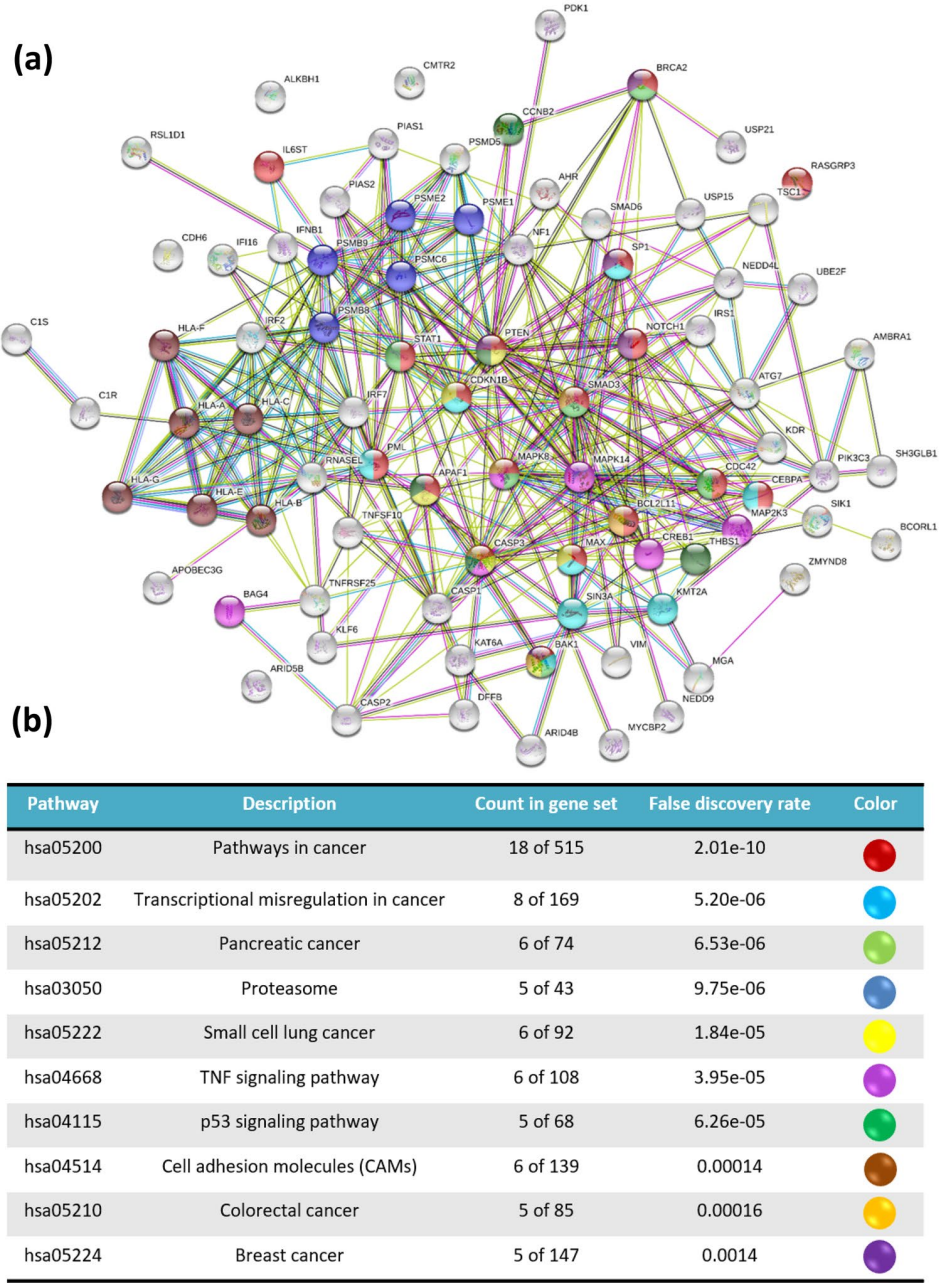
The STRING PPI network based on the KEGG pathways^21–23^. (**a**) The protein–protein interaction of both up/down-regulated DEGs showed a meaningful co-expression. Highlighting nodes by oncogenic signalling pathways illustrated the hub genes. (**b**) The selected oncogenic signalling pathways.

PPI network was visualized with 97 DEGs using STRING database (Fig. 3a). It showed that there was a close relationship among up /down-regulated DEGs. It was observed that there was a highly positive co-expression relationship between PTEN, SMAD3, SP1, CASP3, MAPK8, CDKN1B, CREB1, STAT1, PSMB8, PSMB9, and MAPK14. Moreover, there was a clear association between HLA-F, HLA-A, HLA-C, IRF2, PSMB8, PSMB5, and PSMB8. To highlight the master regulator of the oncogenic pathway, any cancer-related signalling pathway was selected by KEGG (Fig. 3b)^21–23^. As it is clear, there is a strong correlation among the highlighted genes.

### Identification of hub DEGs through network analysis

**Figure 4:**
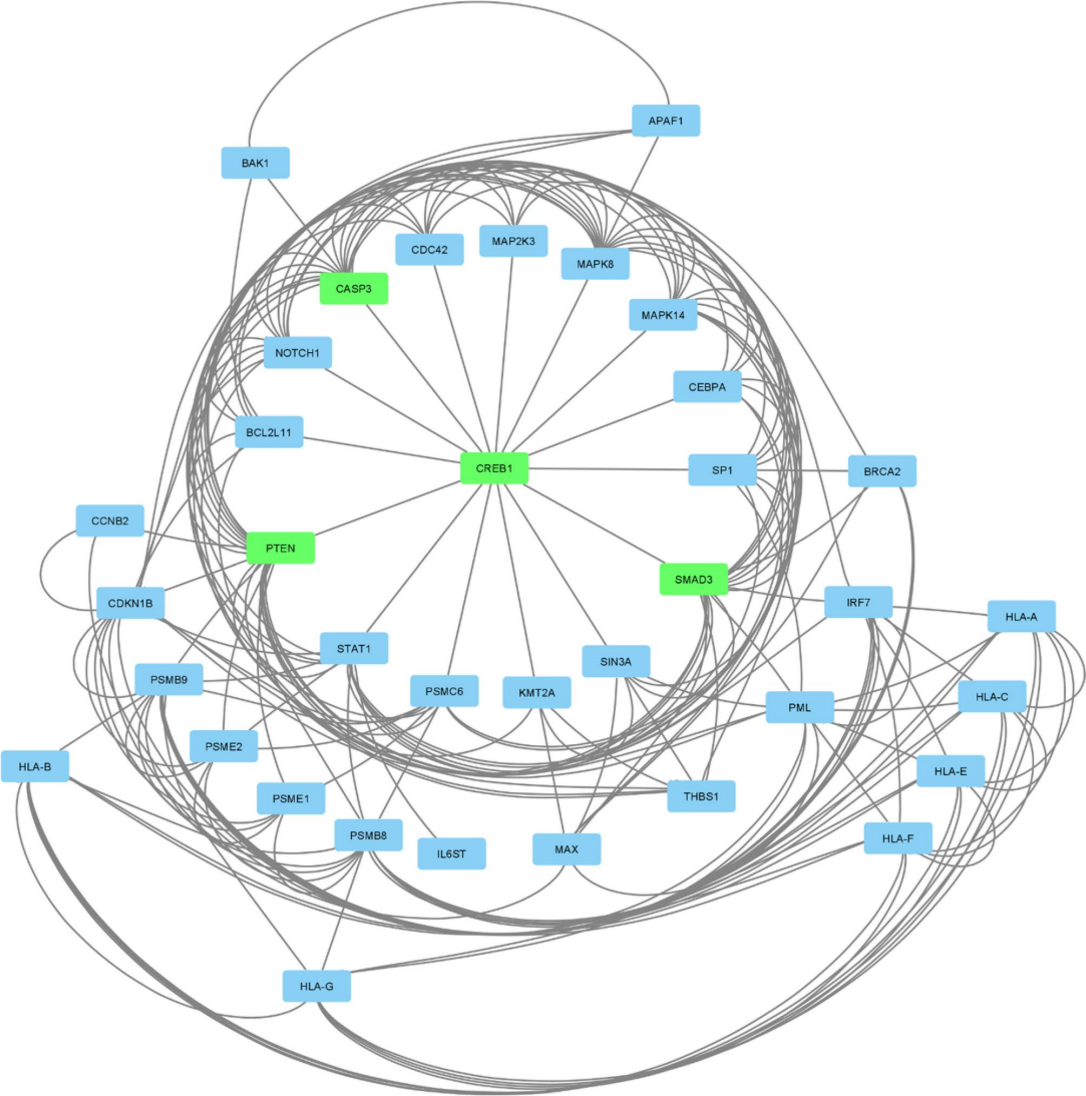
The Cytoscape network indicated hub genes. The yfile radial layout of Cytoscape software showed the most significant hub genes in cancer-related DEGs. The four important hub genes are shown in green.

To identify master genes, Cytoscape was used for network analysis. The highlighted genes from Fig. 3 were uploaded in Cytoscape redraw by yfiles redial layout algorithm (Fig. 4).

**Figure 5:**
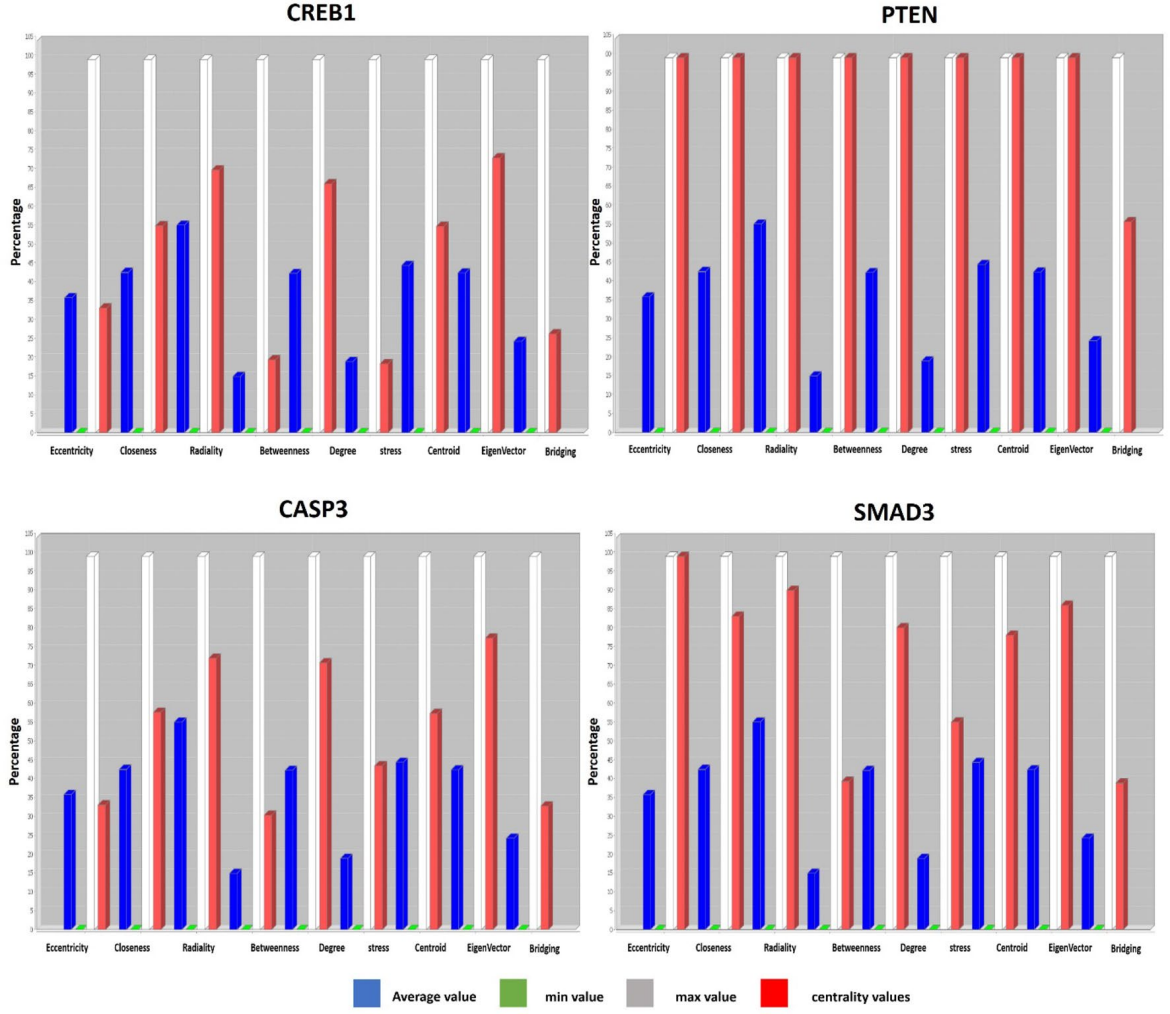
The network analysis of four most important hub genes by Centiscape plug-in. The Centiscape plug-in of Cytoscape software has represented the four most hub genes based on degree and betweenness centrality.

The resulted network categorized DEGs based on their interactions. The 15 most correlative genes with the highest interaction have been ordered as a circle. As it is evident, CREB1 is the only and the main regulator of the most correlative genes. The other layouts of Cytoscape network showed the interplay between less important genes. Cytoscape network analysis tool confirmed the yfiles redial layout. Centiscape plug-in represented *PTEN, SMAD3, CASP3*, and *CREB1* as the most important hub genes based on degree and betweenness centrality (Fig. 5).

### Confirmation of hub genes using TCGA database

**Figure 6:**
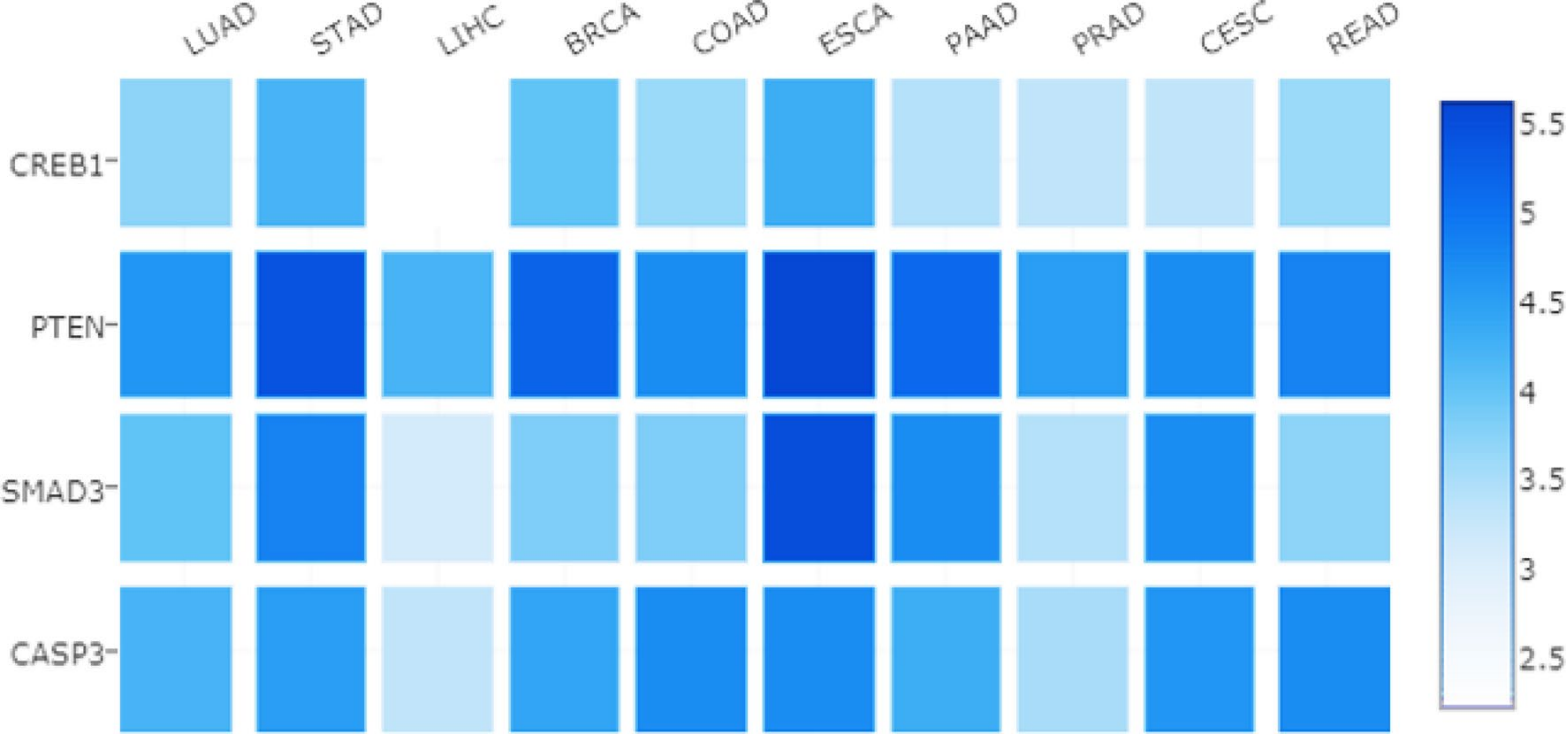
A heatmap of candidate genes in the ten most fatal types of cancer. The GEPIA 2 server has provided the expression levels of candidate genes in the most fatal types of cancer. LUAD (Lung adenocarcinoma), STAD (Stomach adenocarcinoma), LIHC (Liver hepatocellular carcinoma), BRCA (Breast invasive carcinoma), COAD (Colon adenocarcinoma), ESCA (Esophageal carcinoma), PAAD (Pancreatic adenocarcinoma), PRAD (Prostate adenocarcinoma), CESC (Cervical squamous cell carcinoma), READ (Rectum adenocarcinoma).

GEPIA 2 server, which applies RNAseq data, affirmed our candidate genes. The heatmap plot represents the differential expression of *PTEN, CREB1, CASP3*, and *SMAD3* in the ten most fatal malignancies. As it is evident, all four genes have critical role in the selected cancer types (Fig. 6).

Later, using TCGA data, we realized that the expression of PTEN*, CREB1, CASP3*, and *SMAD3* are significantly (|Log FC| ≥ 1 and *p* value < 0.01 as the cut-off criteria) increased in Pancreatic adenocarcinoma (PAAD) (data not shown). Therefore, PAAD was introduced as a possible cancer type following infection with (SARS-Cov-2 family), where the *SMAD3, PTEN, CREB1*, and *CASP3* are overexpressed simultaneously.

**Figure 7:**
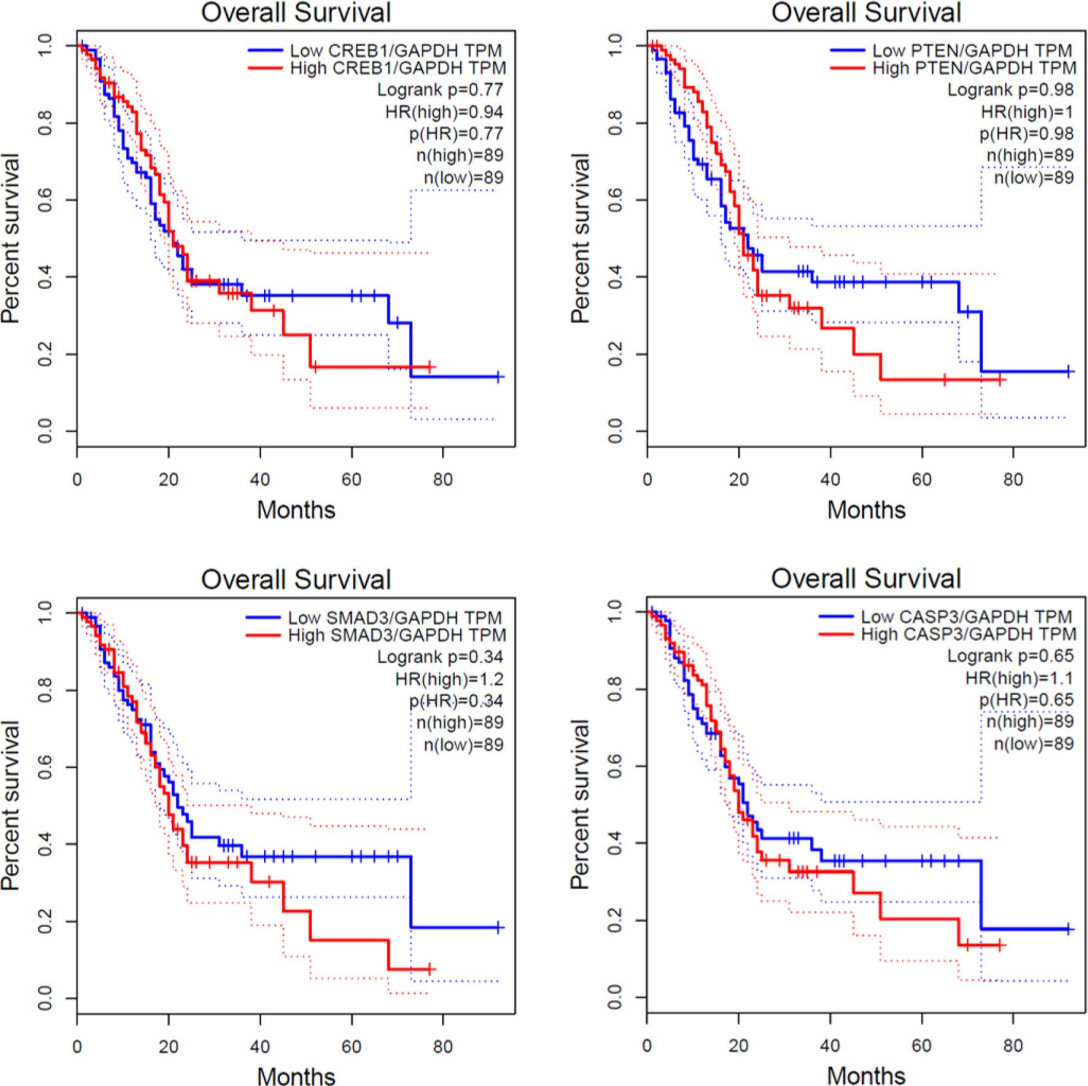
The Kaplan–Meier curves of candidate genes based on TCGA data. The TCGA database illustrated the impact of *CREB, PTEN, SAMD3, CASP3* genes on overall survival rate of patient with pancreatic adenocarcinoma. All four graphs contain blue (low TPM) and red (High TPM) line which normalized by GAPDH.

Then, Kaplan–Meier analysis on PAAD showed an association between the upregulation of *SMAD3*, *PTEN*, *CREB1*, *CASP3*, and decreased patient survivability (Fig. 7). Interestingly, down regulation of all hub genes increased the patient survivability after approximately the 25th month. This information confirmed the relationship between SARS-Cov-2 post-infection and PAAD progression.

### Evaluation and selection of candidate microRNAs

**Figure 8:**
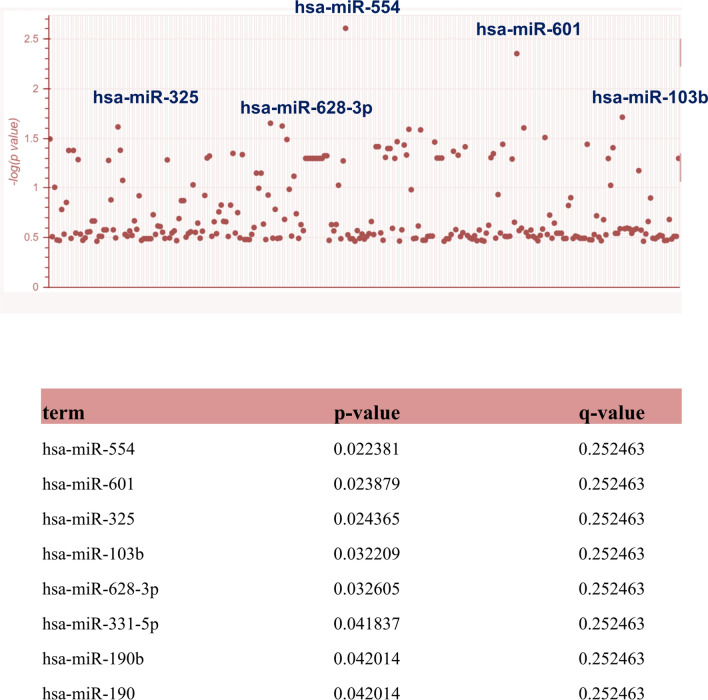
As shown in the picture, the most significant microRNAs associated with the *CREB, PTEN, SMAD3,* and *CASP3* genes were isolated and identified on the Manhattan diagram.

**Figure 9:**
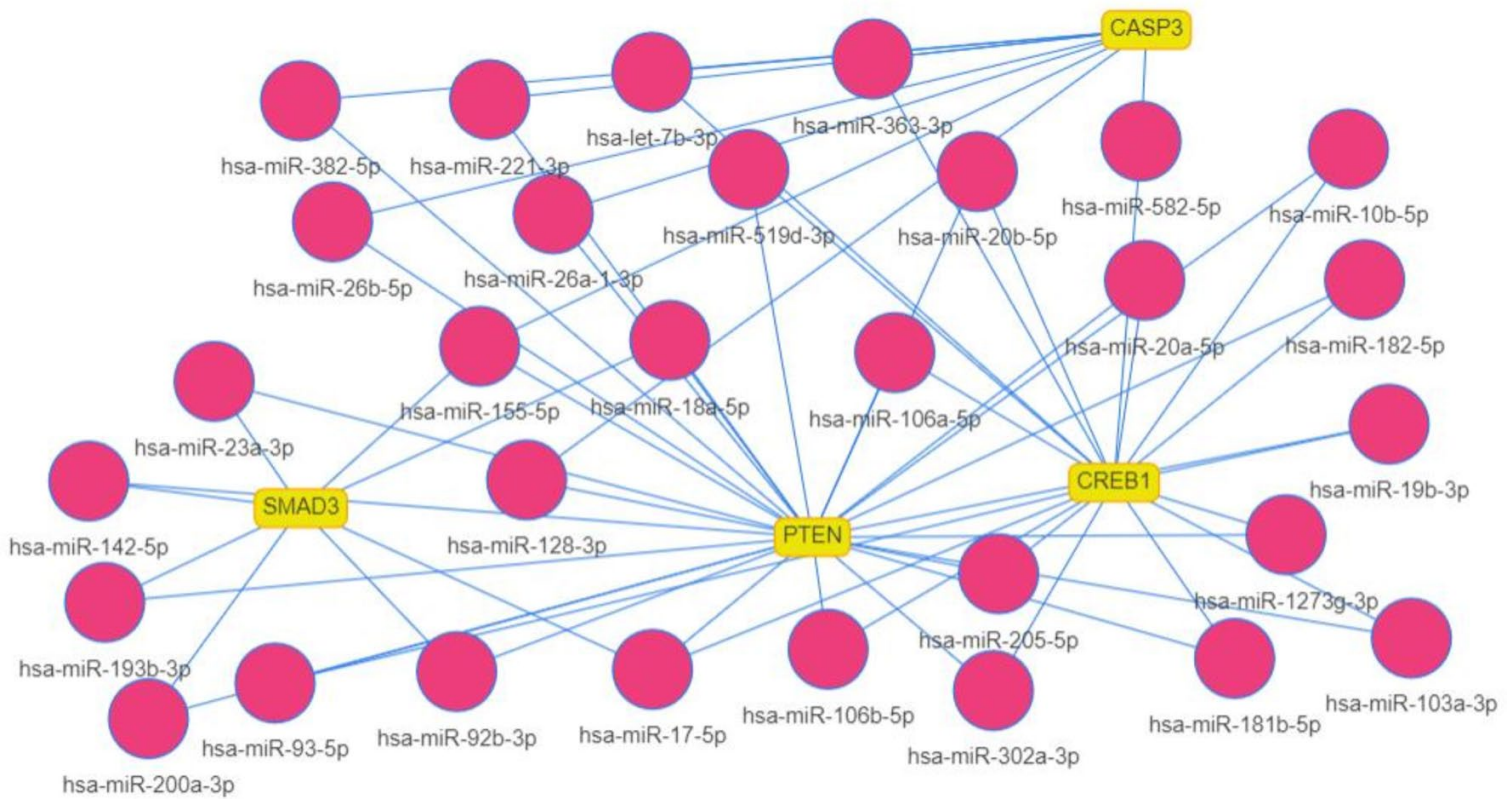
The communication network between *CREB, SMAD3, CASP3*, and *PTEN* is mapped using the MienTurnt database.

In this section, after identifying four proteins CREB, CASP3, SMAD3, and PTEN, we isolated and selected the most relevant microRNAs (Fig. 8). Accordingly, hsa-miR-554, hsa-miR-601, hsa-miR-325, hsa-miR-103b, and hsa-miR-628-3p were observed more clearly than other microRNAs (Fig. 9).

## Discussion

**Figure10:**
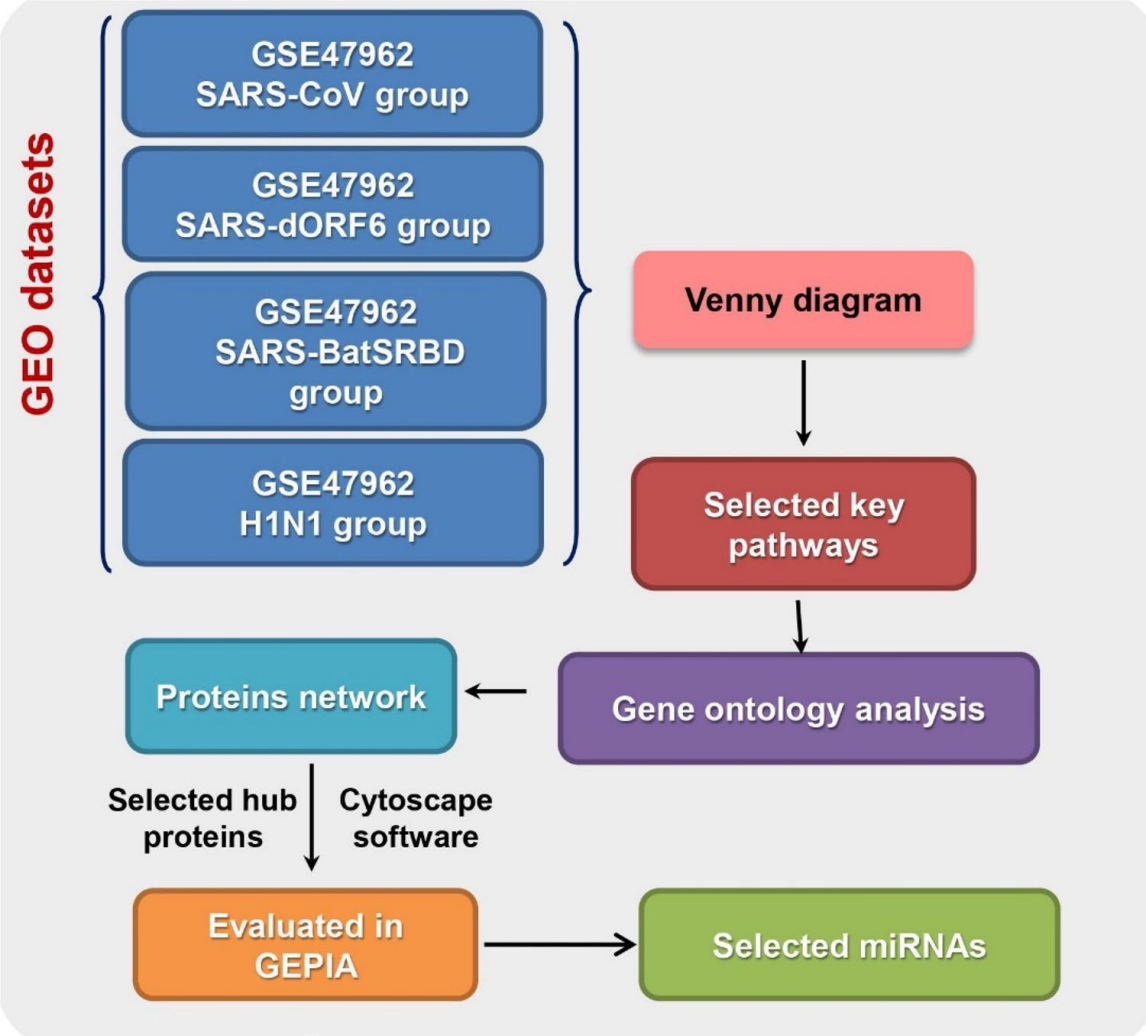
An overview of all the analyses performed in the current study.

Due to the lack of information about the effect of SARS-CoV-2 virus on the expression of different genes, we used datasets related to four SARS-CoV-2 family members in this study (Fig. 10). Gene ontology examination of overexpressed and downregulated genes showed that different biological and functional pathways are involved in infection with these groups of viruses. For example, the molecular function study on upregulated genes showed that the lowest *p* value was related to double-stranded RNA binding "(GO: 0003725). Various studies have highlighted the association between increased expression of RNA-binding proteins and cancer. In general, when an mRNA is transcribed, it undergoes many changes and modifications after transcription. These changes that are made with the help of RNA-binding proteins, can affect the ultimate fate of RNA. The composition of ribonucleoprotein complexes is different and dynamic depending on RNA processing. These proteins can bind to a wide range of RNAs through a variety of domains, such as the double stranded RNA binding domain (dsRBD). Obviously, changes in the expression pattern of RNA-binding proteins can profoundly affect cellular behaviour^24^. Recent studies have shown that altering the expression of these proteins by overexpression of oncogenes and downregulation of tumor suppressor proteins can play an important role in tumorigenesis. For example, the expression of the protein Adenosine deaminases acting on RNA 1 (ADAR1), which has dsRBD motifs, is increased in various cancers such as breast, colon, oesophagus, and etc. A recent study by SUN and colleagues showed that the expression of ADAR1 in pancreatic cancer was significantly higher than normal tissues^25^. Moreover, increased expression of this protein has been associated with poor prognosis of pancreatic cancer. Recently, a high expression of Ribosomal L1 domain containing 1 (RSL1D1), a nuclear protein involved in senescence and regulation of cellular apoptosis, has been associated with poor prognosis of prostate cancer. Although the elevated expression of proteins with the dsRBD motif is not a specific prognosis for cancer, it can serve as a warning sign for the onset and progression of malignancies^26^.

In the present study, the molecular function analysis also showed a decreased expression of actin-binding proteins following infection by SARS-Cov-2 virus family members. Actin-binding proteins include a very wide range of proteins that play a central role in regulating the activity and organization of cytoskeleton actin^27^. In fact, in addition to maintaining cell structure, cell skeleton plays an important role in many cellular biological processes such as cell migration, cytokinesis, endocytosis, and morphogenesis, regulation of gene expression, response to DNA damage, nuclear structure preservation, and nucleocytoplasmic trafficking^28^. Numerous studies underscore the unbalanced expression of actin-binding and regulatory proteins in cancer. For example, decreased expression of Profilin 1 protein as an actin-binding protein has been observed in breast cancer^29^. In addition, decreased expression of Arp2/3 and N-Wasp have been reported in gastric cancer and breast cancer, respectively^30,31^. Therefore, changes in the expression of these proteins after infection with SARS-CoV-2 virus families may result in cancer development. In the present study, PPI network analysis on 97 DEG showed that there was a significant relationship between the overexpressed and downregulated genes. Then, the master genes in the network were identified by Cytoscape. Based on degree and betweenness centrality parameters, four genes *CREB1, PTEN, SMAD3, CASP3* were identified as the hub genes which are discussed in the following paragraphs.

CAMP responsive element binding protein 1 (CREB1) is a transcription factor, which is a part of the leucine zipper family of DNA-binding proteins, participating in several critical biological processes like cell differentiation and proliferation^32^. Overexpression of CREB leads to high cell proliferation, decreased apoptosis and increased angiogenesis^32,33^. CREB is regulated as a transcription factor by phosphorylation and also is activated by Ca2 + and cAMP. This molecule binds to 8 bp palindrome sequences in the promoter and enhancer regions of a number of genes^33^. Based on the phosphorylation pattern, CREB performs specific activities in the cell that alters metabolism, cell cycle, apoptosis, invasion and proliferation^33^. Thus, CREB controls essential cell processes and contributes to immortality and malignancy. CREB-mediated carcinogenesis occurs through over-activation of cAMP-dependent signalling pathways like G-coupled signalling pathways, receptor tyrosine kinase (RTK), JAK/STAT, and consequently secondary signalling pathways^33^. Overexpression of CREB has been observed in various cancer types^33–36^. In addition, overexpression of CREB is associated with clinicopathological parameters such as tumor stage and grade, metastasis, increased recurrence, poor prognosis, and decreased survival rate^32^.

Caspase-3 is a major mediator of apoptosis which is triggered by both intrinsic and extrinsic pathways. This protein is a cysteine protease that targets and breaks down more than 200 proteins that eventually induce apoptosis in cells^37^. Recent reports suggest that caspase-3 may take a role in tumor recurrence and angiogenesis^38^. Liu et al^39^ revealed that in spite of Cas-3 activation, when MCF10A cells were exposed to chemicals and radiation, a remarkable part of the affected cells could survive, emphasizing the Cas-3 contribution in genome instability and cancer development. Caspase-3 also, by a paracrine signalling pathway, causes tumor repopulation after radiotherapy^40^. PC-3 is a precursor of Caspase-3 which is converted to caspase-3 through proteolysis in Asp9, Asp28, and Asp 175^41^. Conversion of PC-3 to caspase-3 is really important in the apoptosis mechanism. Although PC-3 is generally considered to be the inactive zymogenic form of caspase-3, several studies have shown that PC-3 has much less proteolytic activity than caspase-3 (at least 200-fold weaker), which can sometimes establish other functions. Overexpression of PC-3 has been observed in many cancer types^41^.

Following the discovery of membrane-bound TGF-β receptors and their role in proliferation, differentiation and apoptosis, a group of transcription factors known as Smads were introduced as the main mediators of TGF-β signalling. Among these, Smad2 and Smad3 have a more pivotal role in regulating TGF-β function. Due to frequent interaction pattern, these two proteins can perform similar functions in various signalling pathways. It is reported that Smad3 is upregulated in several malignancies that highlights role of this protein in cellular homeostasis^42–44^.

In the present study, we surveyed the expression pattern of four selected hub genes in 10 common fatal cancers. Based on the TCGA database results, the expression levels of CREB1, PTEN, SMAD3, and CASP3 genes are up-regulated in the pancreatic adenocarcinoma. According to the reports, angiotensin-converting enzyme 2 (ACE2) as the main receptor of the SARS-CoV-2 virus is highly expressed on the cell surface of pancreatic cells including exocrine glands and pancreatic islets, making these cells an excellent target for the virus^45^. In a study conducted by Liu et al., the serum levels of amylase and lipase in 121 patients with COVID-19 were measured. According to their results, 1.85% of patients with mild forms of the disease showed high levels of amylase and lipase in their sera, while 17.91% and 16.41% of patients with severe form of COVID-19 exhibited high serum levels of amylase and lipase, respectively. Furthermore, in some of the patients, changes in the pancreas morphology such as increased size of pancreas and tissue damage were evident^46^. These results suggest that pancreatic tissue damage following infection with the SARS-CoV-2 virus may increase the risk of pancreatic cancer through upregulation of genes involved in cancer development. Further studies can provide more detailed information in this area.

## Methods

### Microarray datasets,search strategy and data preparation

COVID-19 related datasets were explored from GEO database (https://www.ncbi.nlm.nih.gov/geo/). The search strategy was (“human” AND “SARS-CoV” AND “epithelium”) and (“homo sapience” AND “COVID-19” AND “epithelium”). Finally, GSE47962 has been selected among the filtered results. GSE47962 was conducted through GPL6480 platform (Agilent-014850 Whole Human Genome Microarray 4 × 44 K G4112F) which consisted of 81 human airway epithelium cells (HAE) infected with SARS-Cov, 21 infected with HAE H1N1, and 32 control samples (PMC372391). Using GEO2R tool, we normalized the high and low expression gene clusters between the viral strains with human airway epithelium cells. Then, the gene expression profiles were collected separately in an excel file. In this section, *p* ˂ 0.05 was considered for the selection of gene clusters.

### Identification of differentially expressed genes (DEGs)

DEGs were screened among defined groups (SARS-CoV, SARS-dORF6, SARS-BatSRBD, and H1N1). In this study, *p* < 0.05 was considered statistically significant. Common up/down-regulated DEGs among four groups were highlighted by Venny 2.1.0 software (http://bioinfogp.cnb.csic.es/tools/venny/).

### Pathway enrichment analysis

Enrichr, an online software tool for gene functional annotation, has been used to investigate KEGG (Kyoto encyclopedia of genes and genomes) enrichment pathway (http://amp.pharm.mssm.edu/Enrichr/)^21–23^. Official gene symbols of common upr/down-regulated DEGs among four groups were used to perform enrichment analysis. ShinyGO v0.61 was used to highlight signalling pathways regulated by DEGs and its importance in cancer development and viral infection (http://bioinformatics.sdstate.edu/go/).

### Gene ontology (GO) investigation

The GO annotation of the common up/down-regulated DEGs was fulfilled through Enrichr and ShinyGO v0.61 software. The biological process, which is mediated by viral infection and cancer development common genes was highlighted by ShinyGO tool.

### Protein–protein interactions (PPI) network analysis

Search Tool for the Retrieval of Interacting Genes (STRING v.11) online tool was applied to predict the functional interactions of proteins (https://string-db.org/). The upregulated genes with a significant role in both viral infection and cancer development were uploaded in the STRING tool. Both known and predicted PPIs were highlighted. To identify the master regulator of cancer, cancer-related signalling pathways were highlighted. The selected genes were imported to Cytoscape (version 3.8.0) with the CentiScape plugin for further analysis and PPI network visualization.

### TCGA and GTEx analysis

GEPIA2 server was used to evaluate the possible relationship between candidate genes and cancer development (http://gepia2.cancer-pku.cn/). The hub genes selected by CentiScape plugin were uploaded on the GEPIA2 server. The heatmap plot showed the differential expression of the introduced hub genes in the ten most fatal types of cancer. Moreover, Kaplan–Meier curve was used to indicate the overall survival.

### Nominate suitable microRNAs

In this section, after finalizing and selecting the genes and candidate proteins, we uploaded the genes to the Enrichr database to evaluate and select the genes-related microRNAs. To aim this, we used the Targetscan library. The Appyter section of the Enrichr database was applied to plot Manhattan. Eventually, the *p* value ˂0.05 was considered significant.

## Conclusion

This research showed that infection with the SARS-CoV family may increase the risk of the cancer development by altering the expression of various oncoproteins. Our findings suggest the pancreatic adenocarcinoma as the most possible malignancy occurring after sever infection with SARS-CoV family.

## Abbreviations

ACE2: Angiotensin-converting enzyme 2
ADAR1: Adenosine deaminases acting on RNA 1
ARP2/3: Actin-related proteins-2/3
BP: Biological process
CASP3: Caspase 3
CREB1: CAMP responsive element binding protein 1
DEGs: Differentially expressed genes
dsRBD: Double stranded RNA binding domain
GEO: Gene expression omnibus
GEPIA: Gene expression profiling interactive analysis
GO: Gene ontology
GTEx: The genotype-tissue expression
HAE: Human airway epithelium cells
KEGG: Kyoto encyclopedia of genes and genomes
Log (FC): Log fold change
MF: Molecular function
NSCLC: Non-small cell lung cancer
Nsp15: Nonstructural protein 15
N-WASP: Neural wiskott-aldrich syndrome protein
PAAD: Pancreatic adenocarcinoma
PPI: Protein–protein interactions
pRb: Retinoblastoma-associated protein
PTEN: Phosphatase and tensin homolog
RNASeq: RNA sequencing
ROS: Reactive oxygen species
RSL1D1: Ribosomal L1 domain containing 1
RTK: Receptor tyrosine kinase
SARS-CoV: SARS-associated coronavirus
SARS-CoV-2: Severe acute respiratory syndrome coronavirus 2
SARS: Severe acute respiratory syndrome
SMAD3: Mothers against decapentaplegic homolog 3
STRING: Search tool for the retrieval of interacting genes
TCGA: The cancer genome atlas
TGF-β: Transforming growth factor beta
TPM: Transcripts per million
TMP1: Tropomyosin 1 alpha
